# Outage Analysis of Unmanned-Aerial-Vehicle-Assisted Simultaneous Wireless Information and Power Transfer System for Industrial Emergency Applications

**DOI:** 10.3390/s23187779

**Published:** 2023-09-09

**Authors:** Aleksandra Cvetković, Vesna Blagojević, Jelena Anastasov, Nenad T. Pavlović, Miloš Milošević

**Affiliations:** 1 Faculty of Mechanical Engineering, University of Niš, 18000 Niš, Serbia; nenad.t.pavlovic@masfak.ni.ac.rs (N.T.P.); milos.milosevic@masfak.ni.ac.rs (M.M.); 2 School of Electrical Engineering, University of Belgrade, 11000 Belgrade, Serbia; vesna.golubovic@etf.rs; 3 Faculty of Electronic Engineering, University of Niš, 18000 Niš, Serbia; jelena.anastasov@elfak.ni.ac.rs

**Keywords:** energy harvesting, industrial emergency applications, outage performance, simultaneous wireless information and power transfer, unmanned aerial vehicle

## Abstract

In the scenario of a natural or human-induced disaster, traditional communication infrastructure is often disrupted or even completely unavailable, making the employment of emergency wireless networks highly important. In this paper, we consider an industrial Supervisory Control and Data Acquisition (SCADA) system assisted by an unmanned aerial vehicle (UAV) that restores connectivity from the master terminal unit (MTU) to the remote terminal unit (RTU). The UAV also provides power supply to the ground RTU, which transmits the signal to the end-user terminal (UT) using the harvested RF energy. The MTU-UAV and UAV-RTU channels are modeled through Nakagami-*m* fading, while the channel between the RTU and the UT is subject to Fisher–Snedecor composite fading. According to the channels’ characterization, the expression for evaluating the overall probability of outage events is derived. The impact of the UAV’s relative position to other terminals and the amount of harvested energy on the outage performance is investigated. In addition, the results obtained based on an independent simulation method are also provided to confirm the validity of the derived analytical results. The provided analysis shows that the position of the UAV that leads to the optimal outage system performance is highly dependent on the MTU’s output power.

## 1. Introduction

The advantages and benefits of utilizing unmanned aerial vehicles (UAVs) have attracted significant attention in both the academic and industrial spheres. UAVs have been extensively utilized across various sectors, including surveillance, military operations, health services, etc. [1]. Furthermore, it has been shown that UAVs are applicable for infrastructure monitoring without imposing danger to humans. Namely, in [2], a UAV-assisted setup equipped with cameras and sensors was utilized for railway inspection and monitoring. Also, UAVs have become indispensable in precision agriculture for essential functions such as crop monitoring, plant health assessment, pest and disease detection, and optimization of irrigation and fertilizer applications [3]. The integration of UAVs in industrial systems has great potential due to their possible applications in maintenance, process monitoring and management, as well as manufacture automatization [4]. Due to their rapid response and flexibility, UAVs can provide stable communication or establish temporary communication links in disaster-stricken areas, especially when existing infrastructure has been damaged or destroyed [5].

The performance of various UAV-assisted systems has been analyzed in the scientific literature [5,6,7,8], with the aim of providing satisfactory solutions for communication needs in emergency-saving scenarios. The issues related to optimization of the UAV position and coping with network destruction in a natural disaster, with and without ground base stations, were examined in [5]. The novel cluster-based mechanism for sensor networks aided by UAVs, which enable data collection over shorter propagation paths and thus improve system performance, was proposed in [6]. Authors in [7] focused on a unified framework for a UAV-assisted emergency multihop device-to-device (D2D) network in disaster areas. The presented results showed a performance improvement in terms of the throughput and outage probability achieved by implementing the UAV-assisted wireless coverage approach. The boundaries of UAV technology applications in industrial disasters and other important directives for further research in this field were identified in [8]. Recently, UAV assistance has also been promoted for general Supervisory Control and Data Acquisition (SCADA) architecture in [9,10].

In recent years, the Internet of Things (IoT) systems and applications have rapidly become ubiquitous and the growth in the number of connected devices has brought higher reliability requests, increased data rates, and energy efficiency. Furthermore, the large number of devices in current networks has a significant influence on the changes in the traditional approaches regarding the powering of transmission nodes. Namely, although battery usage is appealing in some scenarios, due to the large number of nodes, this way of providing energy turns out to be impractical. Wireless power supply represents the alternative approach, where the energy in the environment can be harvested from existing sources such as solar, wind energy, and radio-frequency (RF) energy [11,12]. Within the energy harvesting (EH) approach, the harvesting of RF energy has a special feature, i.e., it can be used for simultaneous information and power transfer (SWIPT) [13,14]. Additionally, RF energy can be purposely transferred to the desired node [15,16]. As SWIPT exploits the principle of energy harvesting from RF signals, that carry both information and energy components, it is suitable for implementation in networks with energy-constrained devices, such as low-power communication devices and sensors, allowing them to recharge their batteries while simultaneously performing the communication tasks. In the case when power is intentionally sent to one of the nodes in the system, additional relaying nodes can be incorporated by applying the time-switching (TS) or power-splitting (PS) protocol [17]. In the TS-based concept, the energy harvester timely switches between the EH and the information transmission phases. The device uses the harvested energy for data transmission. In the PS-based SWIPT concept, the received energy is divided into two portions, where the first one is used for energy harvesting, while the remaining is dedicated for information transmission. This approach allows continuous energy harvesting while still maintaining the system ability to transmit data. When the power reduction in the PS protocol is applied, the device can allocate a larger portion of harvested energy for data transmission, therefore mitigating the impact on the data rate. The decrease in the information transmission time in the TS-based protocol leads to the conservation of energy and the decrease in data rate, while in the PS-based protocol, the transmission time remains constant as the power allocated for transmission is subject to adjustment. The optimal power splitting relay-based cooperative selection scheme was analyzed in [18] for the communication in IoT systems.

In the recently published literature, it is considered that UAVs are capable of fulfilling multiple purposes in the areas of IoT, industry, and other wireless communications. Although they can primarily be employed for information gathering [19,20] and information transfer [21], a very important class of functionalities also encompasses enabling the wireless power supply using RF energy [22,23], as UAVs can provide the power supply in areas that are not accessible when conventional approaches or unmovable nodes are used. Consequently, there are a number of published scientific papers that investigate their system performance in the case when a UAV acts as a harvester or supplier [24,25,26,27,28]. In the case when UAV devices have limited energy and therefore constrained duration of the operating time in the air, they can harvest RF energy for battery charging [24]. Moreover, a signal from a UAV can be used as an RF energy source for supplying energy-constrained devices on the ground [25,26,27,28]. The optimization of a UAV’s trajectory or the minimization of the overall energy consumption in various IoT system scenarios was investigated in [25,28]. The outage and error performance of an IoT system with multiple UAV relays using TS and PS energy harvesting relaying protocols were defined in [24]. In addition, the EH method has been extensively investigated in industrial IoT or in networks for industry automation. In [29], the EH was utilized for information and power transfer to the server machine, which forwards information from the data center to multiple destination machines using a reconfigurable intelligent surface (RIS). In addition, in [30], the RIS was also used to maintain communication among satellite and multiple users in the proposed relay network. The extension of conventional communication to the non-terrestrial systems with/without UAV including communication with satellites in order to fulfil the power constraints of certain nodes was provided in [31,32,33].

### Motivation and Contribution

In modern industry concepts, it is necessary to provide automatized control functions for the end users such as machines, IoT devices, actuators, and sensors. In most scenarios, information is transferred among communication nodes over wireless channels. The outage performance analysis of dual-hop relaying systems with ground relays used for both information signal retransmission and RF energy harvesting can be found in [34,35,36,37,38], for Nakagami-*m* and Fisher–Snedecor fading environments. In our work, we extend the number of communication nodes in accordance with the specific application for industrial purposes. The scenario under consideration corresponds to the industrial SCADA architecture, which consists of the master terminal unit (MTU), the remote terminal unit (RTU), and the user terminal (UT). In a natural or man-made disaster situation, the direct communication between the MTU and the RTU can be corrupted and the powering of the RTU can also be compromised due to the loss of conventional power sources or limited battery life. Since the requirements for the transmission of critical information to the UT node should be met, in this paper, we incorporate a UAV as an additional relay and supplier for the RTU, for such an emergency scenario. In the survey paper about UAV applications [1], a similar system setup was proposed, omitting performance metrics analysis. In [26,27], unified frameworks for charging strategies of a UAV were proposed, which enables SWIPT for IoT nodes or cluster heads with an aim to enhance their further functioning in the networks. With the motivation based on the previously mentioned works, we relate our research to the performance analysis of a SCADA system aided by a UAV, which relays information from the MTU to RTU and enables RTU retransmission to the UT, by supplying the RTU with energy. In the considered analysis, the UAV and the RTU utilize the DF protocol.

In this paper, we provide the system performance analysis and derive the expressions of the probability of outage and the system throughput. We assume that the first link and the second link are Nakagami-*m* fading channels, which represent channel modeling widely utilized in the literature for communication links between the ground nodes and UAV. The third link, RTU-UT, relates to D2D communication over short distances and thus is modeled as a Fisher–Snedecor fading channel. To the best of the authors’ knowledge, the outage performance analysis of Nakagami-*m*/Nakagami-*m*/Fisher–Snedecor relaying systems with the UAV that is employed both as a relay and power supplier for the RTU node has not been previously reported.

This paper’s objectives and main contributions are as follows:

(1) We investigate an industrial SCADA system, assisted by a UAV in a hazardous disaster scenario when the RTU is disconnected from the MTU and also left without power supply.

(2) The novel outage probability and throughput expressions are derived for the considered multihop Nakagami-*m*/Nakagami-*m*/Fisher–Snedecor relaying system, with a UAV employed as a DF relay and energy supplier for RTU.

(3) The outage performance and end-to-end system throughput analysis is carried out, in detail, aiming to adjust the UAV’s relative position above the MTU-RTU link and the amount of supplied power in relation to other essential parameters.

(4) The important insights in the interplay of environmental parameters are provided, such as the amount of MTU output power or harvesting power as well as the UAV position in relation to other communication units, with the aim of enhancing system reliability.

(5) The numerical results based on analytical expressions are provided and compared with simulation results based on developed Monte Carlo simulation model, in order to demonstrate the validity of the derived analytical expressions. Based on the obtained results, additional conclusions concerning the impact of the system and channel model on outage performances are derived.

In brief, this paper is structured as follows. In Section 2, we present the statistical characterization of communication links and describe the RF energy harvesting protocol employed at the RTU. In Section 3, we derive the analytical expression for the outage system performance, whereby part of the mathematical derivation of the performance is provided in detail in the Appendix A. Numerical and simulation results are presented and discussed in Section 4. The conclusion is given in Section 5.

## 2. System and Channel Model

**Figure 1:**
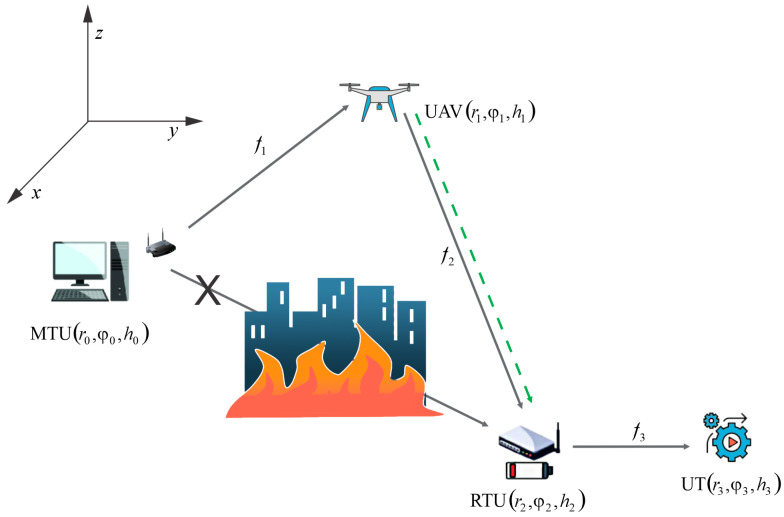
UAV-assisted industrial system for emergency applications.

We consider an industrial-service communication system illustrated in Figure 1, as a solution for the natural disaster scenarios. The traditional communication infrastructure is often disrupted or even completely unavailable due to natural or human disasters (such as earthquakes, floods, bushfires, and tornadoes), demanding the employment of emergency wireless networks. We consider a general system model that can be applied as a SCADA system, in the scenario when it is disabled due to destruction or inefficiency of the existing infrastructure. SCADA, as an important part of industrial systems, consists of an MTU, an RTU, and an end user terminal [9,10], where the RTUs are used at remote destinations and are usually placed in outdoor inaccessible environments. SCADA architecture is utilized for monitoring and management of industrial processes. Therefore, the MTU can send information to the RTU to provide an emergency shutdown of the process, to prevent hazardous situations by starting or stopping pumps or adjusting the speed of pumps, and to regulate the flow of fluids or gases by opening/closing valves [9]. Namely, the industrial control functions are performed by using the communication link, which consists of the MTU-RTU and the RTU-UT hops, but according to the predicted scenario, the communication between the master and the remote unit is disrupted. In addition, the RTU is an energy-limited device left without conventional power supply due to disaster conditions. In such a scenario, the UAV is employed as a relay for information transfer between the MTU and the RTU. It also serves as an energy supplier for the RTU, thus enabling data transmission from the MTU to the end user.

### 2.1. Channel Model

According to a wireless model of propagation [39], the received signal is a sum of multipath delayed components mostly caused by reflection, diffraction, and scattering propagation mechanisms. Consequently, the signal level at the receiver is a random variable and should be statistically determined by the corresponding probability density function (PDF) in order to evaluate the important system performance metrics.

We adopt the assumption that the multipath propagation over communication links between the UAV and the ground nodes is modeled using the Nakagami-*m* distribution, regarding that it is convenient for describing both line-of-sight and non-line-of-sight channel conditions. The received signal envelopes in the MTU-UAV, the UAV-RTU, and the RTU-UT links are denoted by *f*_1_, *f*_2_, and *f*_3_, respectively. Therefore, the channel power gains of the MTU-UAV and the UAV-RTU links are denoted by $g_{1}={\left|f_{1}\right|}_{}^{2}$ and $g_{2}={\left|f_{2}\right|}^{2}$, respectively, and can be statistically characterized by the following Gamma PDFs [39]:

(1) $$p_{g_{i}}\left(\gamma \right)=\frac{1}{\Gamma \left(m_{i}\right)}{\left(\frac{m_{i}}{{\bar{\gamma }}_{i}}\right)}^{m_{i}}\gamma^{m_{i}-1}e^{-\frac{m_{i}}{{\bar{\gamma }}_{i}}\gamma },\;i=1,2,$$

where Γ (·) denotes the Gamma function ([40], (8.310)), *m_i_* denotes the multipath fading parameter, which depends on the signal propagation environment; and ${\bar{\gamma }}_{i}=Ε\left[\gamma_{i}\right]$, *i* = 1, 2.

The communication between the RTU and the UT is typical D2D communication, characterized by relatively small link distances. Accordingly, the RTU-UT link can be described by the Fisher–Snedecor F composite channel, which is proposed as the most appropriate fit model to empirical data of the D2D wireless communication [41,42]. The channel power gain of the RTU-UT link can be denoted by $g_{3}={\left|f_{3}\right|}_{}^{2}$ and the corresponding PDF is then formulated as [41]

(2) $$p_{g}{}_{{}_{3}}\left(x\right)=\frac{{\left(\frac{m_{3}}{m_{s3}{\bar{\gamma }}_{3}}\right)}^{m_{3}}}{B\left(m_{3},m_{s3}\right)}\frac{x^{m_{3}-1}}{{\left(\frac{m_{3}x}{m_{s3}{\bar{\gamma }}_{3}}+1\right)}^{m_{3}+m_{s3}}},$$

where *B*(·,·) denotes the Beta function ([40], (8.380)), ${\bar{\gamma }}_{3}=E\left[\gamma_{3}\right]$ is the average power gain over the RTU-UT link; while *m*_3_ and *m*_s3_ denote the multipath fading and shadowing shaping parameters, respectively.

### 2.2. System Model

We assume that the UAV is utilized to establish the communication from the MTU to the RTU in the case when the direct MTU-RTU link connection is not achievable, and to enable the powering of the RTU. For the considered industrial application of the UAV, its communication requirements must comply with the existing regulative framework provided in [43]. The UAV and the RTU employ the DF relaying scheme to provide transmission of the signal to the end user terminal. Also, the UAV enables energy for the RTU following the PS protocol. We consider the SWIPT protocol based on PS, as it leads to a smaller data rate loss when the same parts of power and time are applied for harvesting in PS and TS protocols, respectively [44].

**Figure 2:**
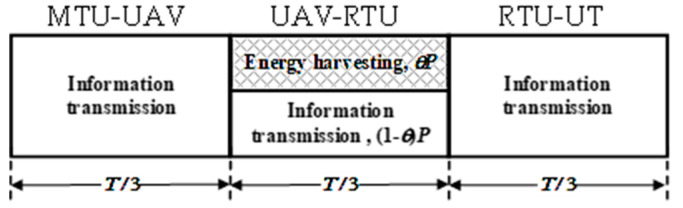
The frame structure during the period *T*.

In the intended communication, the entire time frame period, *T*, is divided into three equal time slots, as shown in Figure 2. The first time slot is used for the MTU-UAV information transmission, the third slot is used for the RTU-UT information transmission, while the second time slot is determined for both the UAV-RTU information transmission and the energy harvesting. During the second time slot, the part of the total power *P* of the received signal, *θ* × *P*, is utilized for the harvesting, while the rest of the power (1 − *θ*) × *P* is dedicated to the UAV-RTU information transmission. The parameter *θ* (0 ≤ *θ* ≤ 1) denotes the power-splitting factor.

Let us assume that the MTU transmits signal *x*_1_ with the power *P_S_*. The received signal at the UAV is then given by

(3) $$y_{1}=\sqrt{\frac{P_{S}}{d_{1}^{\delta_{1}}}}f_{1}x_{1}+n_{1},$$

where *δ*_1_ is the path loss exponent of the first link, MTU-UAV; *n*_1_ denotes the additive white Gaussian noise (AWGN) at the UAV; and *d*_1_ is the distance between the MTU and the UAV. Further, the received signal-to-noise ratio (SNR) at the UAV has the following form:

(4) $$\gamma_{1}=\frac{P_{S}{\left|f_{1}\right|}^{2}}{d_{1}^{\delta_{1}}\sigma_{1}^{2}}=\frac{P_{S}}{d_{1}^{\delta_{1}}\sigma_{1}^{2}}g_{1}$$

where $\sigma_{1}^{2}$ denotes the variance of the AWGN.

The UAV decodes and re-encodes the received signal and transmits signal *x*_2_ with power *P_UAV_*. Thus, the received signal at the RTU, based on the PS protocol, can be expressed as

(5) $$y_{2}=\sqrt{\frac{(1-\theta )P_{UAV}}{d_{2}^{\delta_{2}}}}f_{2}x_{2}+n_{2},$$

where *d*_2_ is the distance between the UAV and the RTU, *δ*_2_ is the corresponding path loss exponent, and *n*_2_ is the AWGN at the RTU. Accordingly, the received SNR at the RTU can be defined as

(6) $$\gamma_{2}=\frac{(1-\theta )P_{UAV}}{d_{2}^{\delta_{2}}\sigma_{2}^{2}}g_{2},$$

where $\sigma_{2}^{2}$ is the variance of the AWGN.

Relying on the PS protocol, the remaining part of the available power is intended for the energy supply of the RTU battery. The total harvested energy by RTU (at the end of the second time slot) can be calculated as

(7) $$E_{H}=\eta \frac{\theta P_{UAV}g_{2}}{d_{2}^{\delta_{2}}}\frac{T}{3},$$

where 0 < *η <* 1 is the energy conversion efficiency.

As the total amount of harvested energy is used for the further transmission, the corresponding transmit power of the RTU can be determined as

(8) $$P_{R}=\frac{E_{H}}{T/3}=\frac{\eta \theta P_{UAV}}{d_{2}^{\delta_{2}}}g_{2},$$

and the received signal at the end UT can be formulated as

(9) $$y_{3}=\sqrt{\frac{P_{R}}{d_{3}^{\delta_{3}}}}f_{3}x_{3}+n_{3}.$$

In addition, based on (8) and (9), the received SNR at the end UT is given as

(10) $$\gamma_{3}=\frac{\eta \theta P_{UAV}}{d_{2}^{\delta_{2}}d_{3}^{\delta_{3}}\sigma_{3}^{2}}g_{2}g_{3}.$$

In Equations (9) and (10), *d*_3_ denotes the distance between the RTU and the end UT, *δ*_3_ is the corresponding path loss exponent, while *n*_3_ is AWGN with the variance $\sigma_{3}^{2}$ at the end user terminal.

## 3. Outage Performance

Statistically, the outage probability is defined as the probability that the instantaneous SNR falls below predefined threshold, *γ_th_*. The outage threshold, *γ_th_*, represents the SNR value that is a boundary between correct system functioning and the system outage. It depends on the specific application and system parameters, such as the modulation format, implementation of the receivers, and bit rates.

The system under consideration will be in outage (or will not function correctly) if any of the three communication links is in outage. Thus, the outage performance of the overall system can be calculated as

(11) $$\begin{array}{c} P_{out}=\mathrm{Pr}\left\{\gamma_{1}\leq \gamma_{th}\right\}+\mathrm{Pr}\left\{\gamma_{2}\leq \gamma_{th}\right\}\mathrm{Pr}\left\{\gamma_{1}>\gamma_{th}\right\}\\ +\mathrm{Pr}\left\{\gamma_{1}>\gamma_{th}\right\}\mathrm{Pr}\left\{\gamma_{3}\leq \gamma_{th},\gamma_{2}>\gamma_{th}\right\}, \end{array}$$

where Pr{·} denotes the probability.

As the variable *γ*_1_ is independent of the random variables *γ*_2_ and *γ*_3_, the probabilities $\mathrm{Pr}\left\{\gamma_{1}\leq \gamma_{th}\right\}$ and $\mathrm{Pr}\left\{\gamma_{1}>\gamma_{th}\right\}$ in (11) can be determined as

(12) $$\mathrm{Pr}\left\{\gamma_{1}\leq \gamma_{th}\right\}=\mathrm{Pr}\left\{g_{1}\leq \frac{d_{1}^{\delta_{1}}\sigma_{1}^{2}}{P_{S}}\gamma_{th}\right\}=F_{g_{1}}\left(\frac{d_{1}^{\delta_{1}}\sigma_{1}^{2}}{P_{S}}\gamma_{th}\right),$$

and

(13) $$\mathrm{Pr}\left\{\gamma_{1}>\gamma_{th}\right\}=1-\mathrm{Pr}\left\{\gamma_{1}\leq \gamma_{th}\right\}=1-F_{g_{1}}\left(\frac{d_{1}^{\delta_{1}}\sigma_{1}^{2}}{P_{S}}\gamma_{th}\right),$$

where $F_{g_{1}}\left(.\right)$ is the cumulative distribution function (CDF) of the Gamma variable. Relying on the PDF expression in (1), the CDF can be expressed as [39]

(14) $$F_{g_{i}}\left(\gamma \right)=1-\frac{\Gamma \left(m_{i},\frac{m_{i}}{{\bar{\gamma }}_{i}}\gamma \right)}{\Gamma \left(m_{i}\right)},\;i=1,2.$$

Further, recalling (6), the probability $\mathrm{Pr}\left\{\gamma_{2}\leq \gamma_{th}\right\}$, can be defined as

(15) $$\mathrm{Pr}\left\{\gamma_{2}\leq \gamma_{th}\right\}=\mathrm{Pr}\left\{g_{2}\leq \frac{d_{2}^{\delta_{2}}\sigma_{2}^{2}\gamma_{th}}{(1-\theta )P_{UAV}}\right\}=F_{g_{2}}\left(\frac{d_{2}^{\delta_{2}}\sigma_{2}^{2}\gamma_{th}}{(1-\theta )P_{UAV}}\right).$$

By introducing $a=\frac{(1-\theta )P_{UAV}}{d_{2}^{\delta_{2}}\sigma_{2}^{2}}$, the instantaneous SNR in (6) becomes $\gamma_{2}=ag_{2}$, and the PDF of *γ*_2_ is defined, following the relation $p_{\gamma_{2}}\left(\gamma \right)=p_{g_{2}}\left(\gamma /a\right)/a$ [45], as

(16) $$p_{\gamma_{2}}\left(\gamma \right)=\frac{1}{\Gamma \left(m_{2}\right)}{\left(\frac{m_{2}}{a{\bar{\gamma }}_{2}}\right)}^{m_{2}}\gamma^{m_{2}-1}e^{-\frac{m_{2}}{a{\bar{\gamma }}_{2}}\gamma }.$$

Finally, by making change $b=\frac{\eta \theta \sigma_{2}^{2}}{(1-\theta )d_{3}^{\delta_{3}}\sigma_{3}^{2}}$ in (10), we obtain $\gamma_{3}=b\gamma_{2}g_{3}$, and the probability $\mathrm{Pr}\left\{\gamma_{3}\leq \gamma_{th},\gamma_{2}>\gamma_{th}\right\}$ can be rewritten as

(17) $$\mathrm{Pr}\left\{\gamma_{3}\leq \gamma_{th},\gamma_{2}>\gamma_{th}\right\}=\mathrm{Pr}\left\{b\gamma_{2}g_{3}\leq \gamma_{th},\gamma_{2}>\gamma_{th}\right\}=\mathrm{Pr}\left\{g_{3}\leq \frac{\gamma_{th}}{b\gamma_{2}},\gamma_{2}>\gamma_{th}\right\}.$$

The analytical derivation of Equation (17) is provided in detail in Appendix A, and the solution can be expressed as its approximate closed form as

(18) $$\begin{array}{l} \mathrm{Pr}\left\{\gamma_{3}\leq \gamma_{th},\gamma_{2}>\gamma_{th}\right\}≅\frac{1}{\Gamma \left(m_{2}\right)\Gamma \left(m_{3}+m_{s3}\right)B\left(m_{3},m_{s3}\right)}{\left(\frac{m_{2}\gamma_{th}}{a{\bar{\gamma }}_{2}}\right)}^{m_{2}}\\ .[{\left(\frac{m_{3}}{m_{s3}{\bar{\gamma }}_{3}b}\right)}^{m_{2}}G_{2,3}^{2,2}\left(\frac{m_{2}m_{3}\gamma_{th}}{a{\bar{\gamma }}_{2}m_{s3}{\bar{\gamma }}_{1}b}|\begin{array}{c} 1-m_{2},1-m_{2}-m_{s3}\\ 0,m_{3}-m_{2},-m_{2} \end{array}\right)\\ -{\left(\frac{m_{3}}{m_{s3}{\bar{\gamma }}_{3}b}\right)}^{m_{3}}G_{3,3}^{2,2}\left(\frac{m_{s3}{\bar{\gamma }}_{3}b}{m_{3}}|\begin{array}{c} \begin{array}{ccc} 1-m_{2}+m_{3}, & 1, & 1+m_{3} \end{array}\\ \begin{array}{ccc} m_{3}, & m_{2}+m_{s3}, & m_{3}-m_{2} \end{array} \end{array}\right)], \end{array}$$

where $G_{p,q}^{m,n}\left(\cdot \right)$ denotes Meijer’s *G* function ([40], (9.301)).

Hence, by substituting the derived equations (Equations (12), (13), (15) and (18)) into (11), we obtain the exact closed-form approximate result for the probability of outage, i.e., the probability of a system being in a failure.

Moreover, we define the maximum data rate, achievable in the channel as a consequence of a deep fading, so-called the outage capacity. The DF system is said to be in outage in the case when the SNR at any of the receive nodes in all three hops is lower than the predetermined threshold, *γ_th_*. For the probability of an outage equal to *P_out_*(*γ_th_*), the normalized capacity is given by the following expression [17]:

(19) $$C_{out}=\frac{1}{3\ln2}\left(1-P_{out}\left(\gamma_{th}\right)\right)\ln\left(1+\gamma_{th}\right).$$

Thus, the achievable throughput *T_out_* is determined as

(20) $$T_{out}=C_{out}.$$

## 4. Numerical Results

In this section, we present numerical results based on the analysis formulated in the previous section and develop an independent Monte Carlo simulation method, with an aim to investigate the impact of various system and channel parameters on the outage performance. Numerical results for the outage probability and achievable throughput are obtained based on derived analytical expressions and are compared with the simulation results obtained using the independent Monte Carlo simulation model. From the obtained results, it can be concluded that the results based on the simulation method and analytical expression are in excellent agreement, showing the accuracy of the developed analysis.

**Figure 3:**
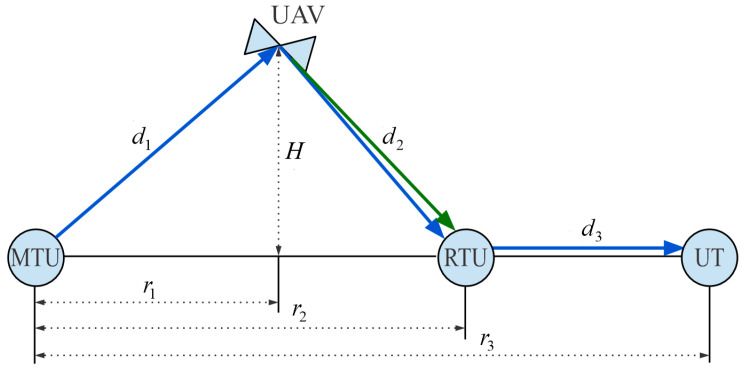
Nodes’ positions in UAV-assisted industrial system for emergency applications.

The location of each network node is determined in the cylindrical coordinate system as MTU(*r*_0_, *φ*_0_, *h*_0_), UAV(*r*_1_, *φ*_1_, *h*_1_), RTU(*r*_2_, *φ*_2_, *h*_2_), and UT(*r*_3_, *φ*_3_, *h*_3_). For the sake of simplicity, the coordinates of the nodes’ positions, for the presented numerical results, are MTU(0 m, 0 rad, 0 m), UAV(*r*_1_, 0 rad, *H*), RTU(400 m, 0 rad, 0 m), and UT(450 m, 0 rad, 0 m). Consequently, the system model can be redrawn as in Figure 3. The distances between nodes *d_i_*, *i* = 1, 2, 3, are defined by the Euclidean norm. Further, the following system parameters are set as *θ* = 0.8, *δ*_1_ = *δ*_2_ = *δ*_3_ = 2.05, $\sigma_{1}^{2}=10^{-5}\mathrm{mW}$, and $\sigma_{2}^{2}=\sigma_{3}^{2}=10^{-7}\mathrm{mW}$. In addition, the fading parameters that describe conditions of channels are set as *m*_1_ = 5 for the MTU-UAV link, *m*_2_ = 2 for the UAV-RTU link, and *m*_3_ = 3.5 and *m*_s3_ = 5 for the RTU-UT link.

According to Figure 3, the specified distances in the MTU-UAV, the UAV-RTU, and the RTU-UT links are $d_{1}=\sqrt{r_{1}^{2}+H^{2}}$, $d_{2}=\sqrt{{\left(r_{2}-r_{1}\right)}^{2}+H^{2}}$, and $d_{3}=\left|r_{3}-r_{2}\right|=50\;m$, respectively.

**Figure 4:**
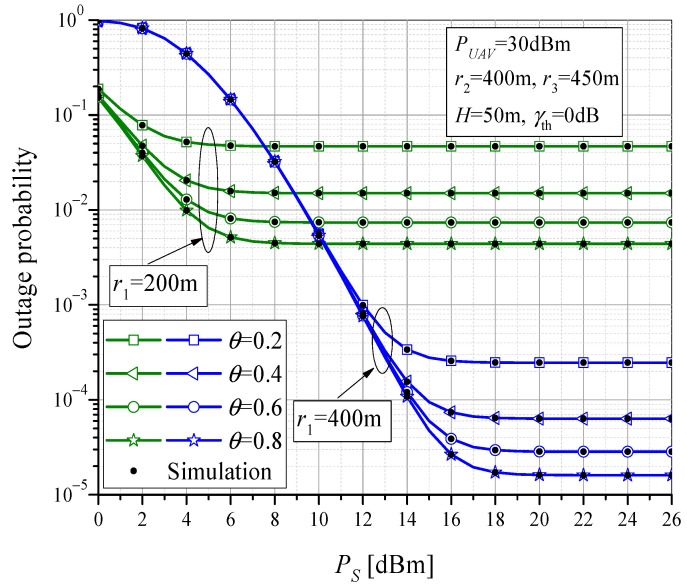
The outage probability vs. MTU output power for various power-splitting factors.

The outage probability versus (vs.) the MTU transmit power *P*_S_ is presented in Figure 4, for different values of power-splitting factor *θ*. The results are obtained for two different values of horizontal distances in the MTU-UAV link, i.e., for *r*_1_ = 200 m and *r*_1_ = 400 m. It can be observed that with the increase in the MTU transmit power, the probability of outage decreases, up to a certain value after which the probability tends to a constant value, i.e., it enters saturation. The further power increase does not improve the outage performance, which can be explained by the fact that the system performance is dominantly determined by the link with the worst conditions and, for high values of *P_S_*, indicates that the rest of the system is interrupted, regardless of the transmit power of the MTU-UAV link. The outage probability is also dependent on the power-splitting factor since higher values of *θ* indicate a greater value of the harvested power at the RTU, consequently leading to the higher RTU transmit power and enhanced system performance. The increase in the horizontal distance component in the MTU-UAV link results in the increase in the total MTU-UAV distance *d*_1_, to higher path losses of the observed link, and, thus, to worse system performance for smaller values of *Ps* (up to 10 dBm). For output power values above *Ps* = 10 dBm, better system performance is achieved when the MTU-UAV distance is larger because the UAV-RTU distance has a lower value, and subsystem UAV-RTU-UT dominantly determines the system performance. For *r*_1_ = *r*_2_ = 400 m, the distance between the UAV and RTU is smallest and equals *H* (as the UAV is directly above the RTU), the path-loss is reduced, and for all analyzed scenarios, the probability of system failure is lower.

**Figure 5:**
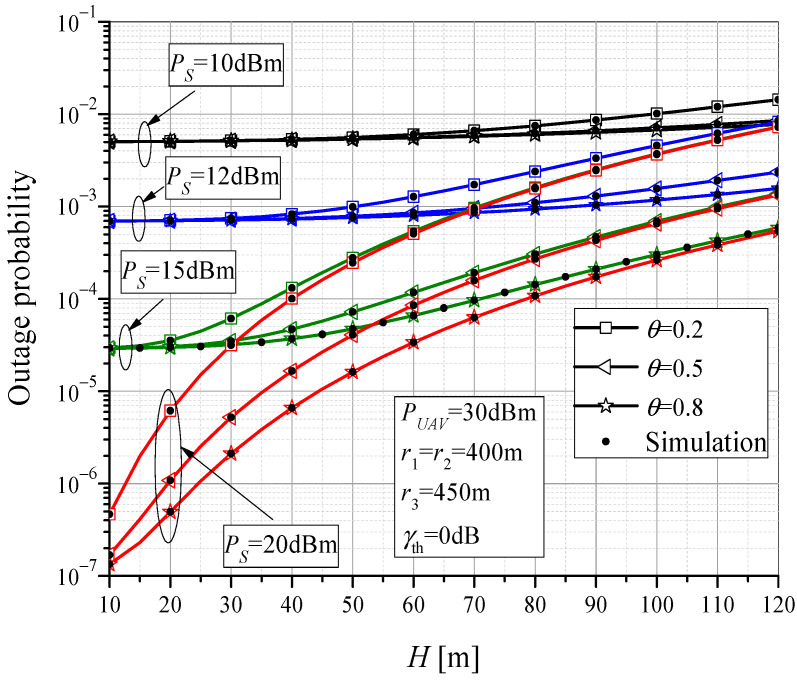
The outage probability vs. UAV altitude for various power-splitting factors.

In Figure 5, the outage performance dependence on the UAV altitude is shown, for the case when the UAV is above RTU (*r*_1_ = *r*_2_ = 400 m). Numerical and simulation results are obtained for different values of MTU transmit power *P_S_* and power-splitting factor *θ*. For lower values of *P_S_*, the MTU-UAV link represents the critical one for the outage, resulting in a higher probability of system failure. In this case, the influence of the amount of harvested energy and the influence of UAV altitude is not significant. By increasing the MTU output power, the probability of an outage event dominantly depends on the failure of the rest of system, and thus the power-splitting ratio has a significant impact on the system outage. The performance improves when the collected energy on the RTU is higher, i.e., when *θ* is larger. In addition, with increasing UAV position height, the outage probability increases due to higher path-loss, and the influence of UAV altitude on the performance is significant.

**Figure 6:**
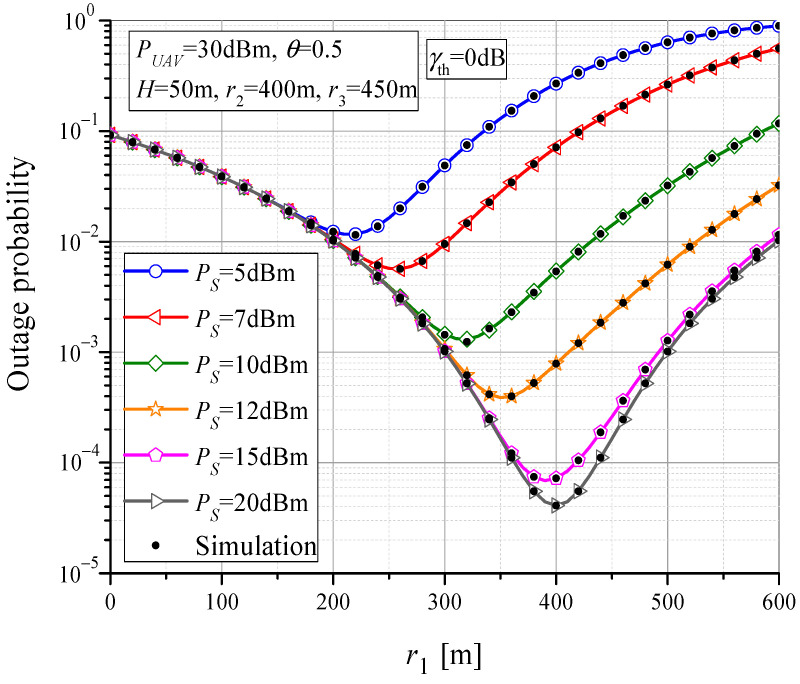
The outage probability vs. MTU-UAV horizontal distance for various values of MTU output power.

The dependence of the outage probability on horizontal MTU-UAV distance for various values of *P_S_* is presented in Figure 6. When the UAV is located closer to the MTU (*r*_1_ < 200 m), the impact of the transmitted MTU power on the outage probability is negligible, and by increasing the distance *r*_1_, the outage probability decreases. For the certain horizontal MTU-UAV distance value, the minimum probability of outage occurs. The outage probability increases with the further increase in *r*_1_. This effect can be intuitively explained by the fact that if the wireless power transfer is applied for the RTU power supply, the best performance is obtained when the UAV is directly above the RTU. However, this fact is valid only for higher values of *P_S_*; thus, the outage of the MTU-UAV link does not affect the overall outage performance. In general, the optimal location of the UAV that contributes to the minimum of the outage probability is located between the MTU and the RTU. For smaller values of *P_S_*, the optimal performance is obtained when the UAV is positioned closer to the MTU, and vice versa. The best outage performance is obtained in the case of high MTU output power, when the UAV is positioned directly above the RTU, which harvests the energy from the UAV.

In Table 1, the values of UAV position *r*_1_ that lead to the minimum outage probability as well as the corresponding outage probability values are listed for various *P_S_* values and the following set of parameters: *η* = 0.5, *γ*_th_ = 0 dB, *P_UAV_* = 30 dBm, and *H* = 50 m. It can be noticed that for the given set of parameters, increasing the MTU power beyond 20 dBm does not lead to a further improvement in system performance. For higher values of MTU output power, the optimal UAV distance *r*_1_ is equal to *r*_2_.

**Figure 7:**
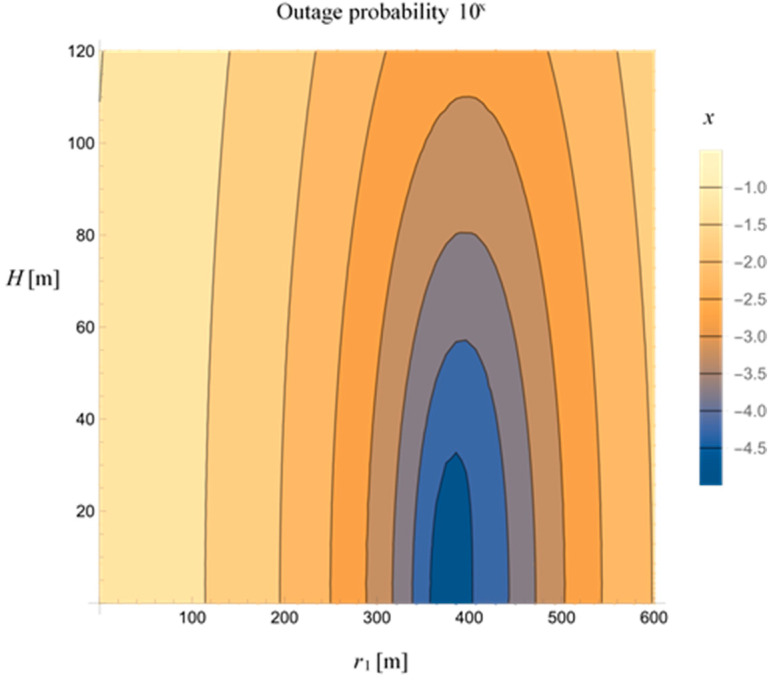
The contour plot of outage probability vs. UAV position *r*_1_ and *H* for *P_S_* = 15 dBm.

The contour plot of the outage probability dependence on the UAV position defined by (*r*_1_, *H*) is presented in Figure 7, for *P_S_* = 15 dBm, *P_UAV_* = 30 dBm, and *θ* = 0.5. The results present a set of values of the UAV height *H* and the MTU-UAV horizontal distance *r*_1_, which lead to the predefined outage probability. When the UAV is positioned at a certain height, changing the MTU-UAV horizontal distance could lead to the predefined outage probability and vice versa. For instance, to obtain an outage probability smaller than 10^−3^, the distance *r*_1_ should be in the range $r_{1}\in \left(300\;m,500\;m\right)$ whereby the height of the UAV can be up to 110 m. To achieve an outage probability smaller than 10^−4^, the *r*_1_ distance should be between 350 m and 450 m, while the maximal UAV’s height *H* can be around 50 m.

**Figure 8:**
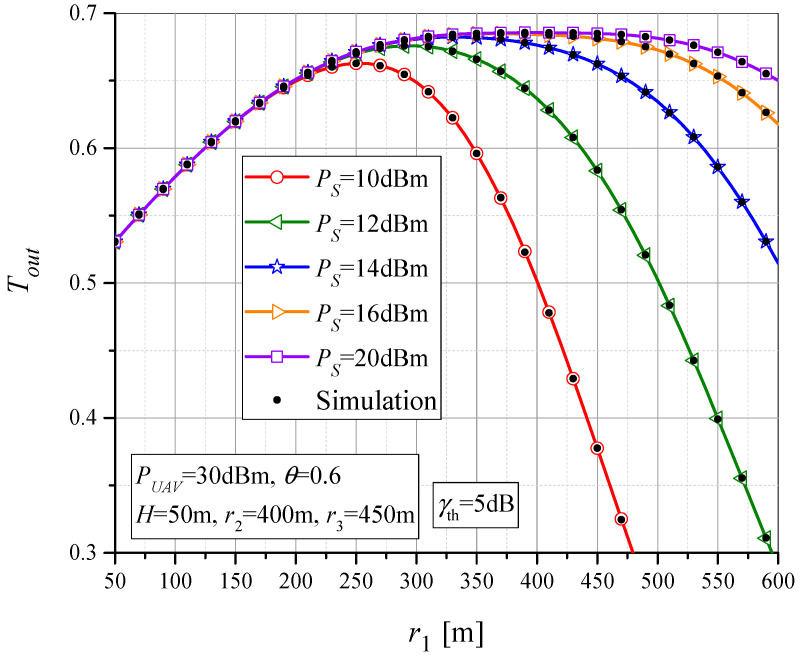
The throughput vs. MTU-UAV horizontal distance for various values of MTU output power.

In Figure 8, the throughput *T_out_* is shown as a function of MTU-UAV horizontal distance, *r*_1_, for parameter values *θ* = 0.7, *H* = 50 m, and *γ_th_* = 5 dB. The results are presented for different values of MTU output power, *P_S_*. It can be noticed that for each value of *P_S_*, there is an optimal UAV position at which maximum throughput is achieved. When the UAV is closer to the MTU, the transmitted MTU power does not affect the throughput and it is determined by the UAV-RTU-UT subsystem. However, as the UAV-MTU distance increases, the throughput also increases due to smaller UAV-RTU distance. At a certain distance *r*_1_, the maximum throughput can be reached. With the increase in MTU transmit power *P_S_*, the value of maximum throughput also increases and it is achieved for higher values of distance *r*_1_. With a further increase in *r*_1_ (beyond the one that maximizes throughput), the throughput decreases and the overall system performance deteriorates.

**Figure 9:**
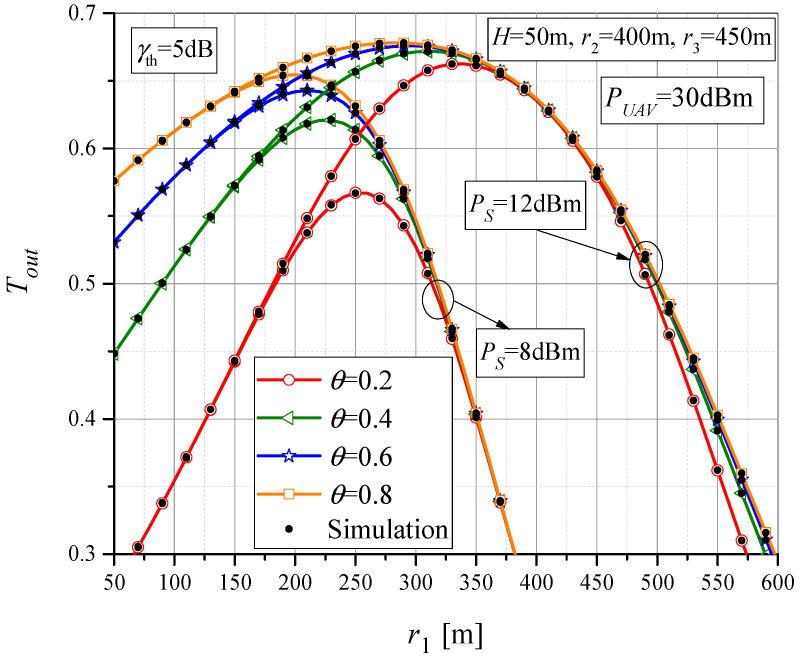
The throughput vs. MTU-UAV horizontal distance for various power-splitting factors.

The throughput dependence on the horizontal MTU-UAV distance for various values of power-splitting factors *θ* is shown in Figure 9. When the UAV position is closer to the MTU, the throughput does not depend on *Ps* (as in Figure 8), but only on the amount of harvested energy at the RTU. With an increase in the power-splitting factor *θ*, the amount of harvested energy at the RTU is larger, which allows the maximum throughput value to be achieved at larger UAV-RTU distances, i.e., at a smaller *r*_1_. Also, when the power-splitting value is smaller, the maximum value of the throughput is smaller. Overall, it is noticeable that the maximum throughput is highly dependent on the position of the UAV, the transmit power of MTU, and the amount of harvested energy.

**Figure 10:**
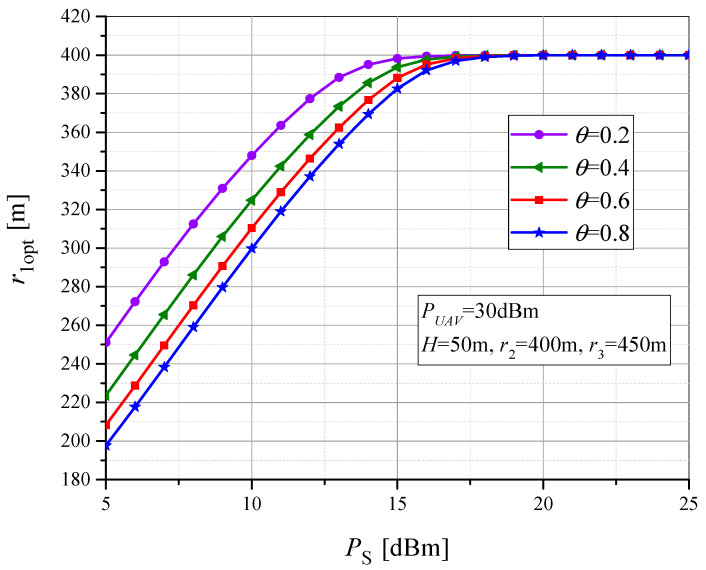
The optimal values of MTU-UAV horizontal distance vs. MTU output power for various power-splitting factor.

Figure 9 shows the dependence of the optimal value *r*_1_ on the power-splitting factor *θ* (and therefore on the amount of collected energy at the RTU). In order to investigate the dependence of the optimal value of the horizontal position between the UAV and the MTU on the power-splitting factor and the MTU transmit power, we present the results in Figure 10. For *P_S_* = 10 dBm and *θ* = 0.8, the optimal horizontal UAV-MTU distance is *r*_1opt_ = 300 m, while for *θ* = 0.2, the optimal value is *r*_1opt_ = 350 m, due to the smaller value of the collected power at the RTU. Therefore, in the case of smaller power-splitting factor values, the optimal position *r*_1opt_ is higher in order to reduce the UAV-RTU distance and corresponding path loss. When *P_S_* > 18 dBm, the optimal MTU-UAV horizontal distance is independent of the power-splitting factor and corresponds to the position when the UAV is above the RTU.

## 5. Conclusions

In this paper, we propose and analyze the outage performance of an industrial system assisted by an unmanned aerial vehicle, which is resilient to the emergency scenario when direct communication between the master terminal unit and the remote terminal unit is disabled or the RTU is left without power supply due to an unpredictable disaster. In the proposed system, the UAV is utilized as both a relay for information communication and as a power supplier for the RTU, which forwards decoded information further to the intended end node. The analytical expressions of the outage event probability and system throughput are derived and the impact of system parameters on the system performance is examined.

The obtained results show that the probability of an outage and the achievable throughput are highly dependent on the position of the UAV relative to the MTU and the RTU, the MTU output power, and the power splitting factor values, i.e., the amount of harvested energy. For lower values of the MTU output power, the UAV should be positioned closer to the MTU. Then, the outage probability depends strongly on the amount of harvested energy and D2D link characteristics. Otherwise, if the MTU power is larger, to ensure that the first link is not in failure, the position of the UAV should be close to the RTU in order to provide the RTU with sufficient output power. It has been shown that the optimal values of the UAV position highly depend on the MTU output power and the amount of harvested energy at the RTU, and these values are calculated for the considered system and channel parameters.

The presented results are useful in the design of an industrial system resilient to the emergency outage scenario, in terms of making an efficient tradeoff between system parameters and the level of outage events, to assure reliable signal transmission.

## Figures and Tables

**Table 1:** Optimal values of *r*_1_ to achieve minimum of outage probability for different *Ps* values.

*P_S_* [dBm]	*r*_1opt_ [m]	P_out_
5	215.10	1.16 × 10^−2^
6	235.81	8.28 × 10^−3^
7	256.70	5.64 × 10^−3^
8	277.42	3.65 × 10^−3^
9	297.60	2.24 × 10^−3^
10	316.90	1.30 × 10^−3^
11	335.10	7.22 × 10^−4^
12	352.02	3.87 × 10^−4^
13	367.50	2.04 × 10^−4^
14	380.99	1.12 × 10^−4^
15	390.99	6.88 × 10^−5^
16	396.47	5.09 × 10^−5^
17	398.75	4.42 × 10^−5^
18	399.58	4.19 × 10^−5^
19	399.86	4.12 × 10^−5^
20	399.95	4.09 × 10^−5^
21	399.98	4.08 × 10^−5^
22	399.99	4.08 × 10^−5^

## Data Availability

Not applicable.

## Appendix A

In order to obtain the analytical form of the overall outage probability, the probability in Equation (17), $\mathrm{Pr}\left\{\gamma_{3}\leq \gamma_{th},\gamma_{2}>\gamma_{th}\right\}$, is expressed as

(A1) $$\begin{array}{l} \;\mathrm{Pr}\left\{\gamma_{3}\leq \gamma_{th},\gamma_{2}>\gamma_{th}\right\}=\int_{\gamma_{th}}^{\infty }F_{g_{3}}\left(\frac{\gamma_{th}}{b\gamma_{2}}\right)p_{\gamma_{2}}\left(\gamma_{2}\right)d\gamma_{2}=\\ =\int_{0}^{\infty }F_{g_{3}}\left(\frac{\gamma_{th}}{b\gamma_{2}}\right)p_{\gamma_{2}}\left(\gamma_{2}\right)d\gamma_{2}-\int_{0}^{\gamma_{th}}F_{g_{3}}\left(\frac{\gamma_{th}}{b\gamma_{2}}\right)p_{\gamma_{2}}\left(\gamma_{2}\right)d\gamma_{2}=I_{1}-I_{2}\;, \end{array}$$

with

(A2) $$I_{1}=\int_{0}^{\infty }F_{g_{3}}\left(\frac{\gamma_{th}}{b\gamma_{2}}\right)p_{\gamma_{2}}\left(\gamma_{2}\right)d\gamma_{2},$$

and

(A3) $$I_{2}=\int_{0}^{\gamma_{th}}F_{g_{3}}\left(\frac{\gamma_{th}}{b\gamma_{2}}\right)p_{\gamma_{2}}\left(\gamma_{2}\right)d\gamma_{2},$$

where $F_{g_{3}}\left(.\right)$ is the CDF of a Fisher–Snedecor *F* variable. The CDF is defined as [42]

(A4) $$F_{g_{3}}\left(\gamma \right)={\left(\frac{m_{3}\gamma }{m_{s3}{\bar{\gamma }}_{3}}\right)}^{m_{i}}\frac{1}{\Gamma \left(m_{3}+m_{s3}\right)B\left(m_{3},m_{s3}\right)}G_{2,2}^{1,2}\left(\frac{m_{3}\gamma }{m_{s3}{\bar{\gamma }}_{3}}|\begin{array}{c} 1-m_{3},1-m_{3}-m_{s3}\\ 0,-m_{3} \end{array}\right).$$

By substituting Equations (A4) and (1) into Equation (A2), the integral *I*_1_ can be rewritten as

(A5) $$\begin{array}{c} I_{1}=\frac{1}{\Gamma \left(m_{3}+m_{s3}\right)B\left(m_{3},m_{s3}\right)}{\left(\frac{m_{3}\gamma_{th}}{m_{s3}{\bar{\gamma }}_{3}b}\right)}^{m_{3}}\frac{1}{\Gamma \left(m_{2}\right)}{\left(\frac{m_{2}}{a{\bar{\gamma }}_{2}}\right)}^{m_{m2}}\\ \;\;\;\;\;\;\;\;\cdot \int_{0}^{\infty }\gamma_{2}^{-m_{3}+m_{2}-1}G_{2,2}^{1,2}\left(\frac{m_{3}\gamma_{th}}{m_{s3}{\bar{\gamma }}_{3}b\gamma_{2}}|\begin{array}{c} 1-m_{3},1-m_{3}-m_{s3}\\ 0,-m_{3} \end{array}\right)e^{-\frac{m_{2}}{a{\bar{\gamma }}_{2}}\gamma_{2}}d\gamma_{2}. \end{array}$$

In order to solve integral in *I*_1_, the exponential function is expressed in the form of Meijer’s *G* function ([46], (01.03.26.0004.01)) as

(A6) $$e^{-\frac{m_{2}}{a{\bar{\gamma }}_{2}}\gamma_{2}}=G_{0,1}^{1,0}\left(\frac{m_{2}}{a{\bar{\gamma }}_{2}}\gamma_{2}|\begin{array}{c} -\\ 0 \end{array}\right),$$

and relying on the argument simplification of Meijer’s *G* function ([46], (07.34.16.0002.01))

(A7) $$G_{2,2}^{1,2}\left(\frac{m_{3}\gamma_{th}}{m_{s3}{\bar{\gamma }}_{3}b\gamma_{2}}|\begin{array}{c} 1-m_{3},1-m_{3}-m_{s3}\\ 0,-m_{3} \end{array}\right)=G_{2,2}^{2,1}\left(\frac{m_{s3}{\bar{\gamma }}_{3}b\gamma_{2}}{m_{3}\gamma_{th}}|\begin{array}{c} 1,1+m_{3}\\ m_{3},m_{3}+m_{s3} \end{array}\right),$$

the integral in Equation (A5) becomes

(A8) $$\begin{array}{c} I_{1}=\frac{1}{\Gamma \left(m_{3}+m_{s3}\right)B\left(m_{3},m_{s3}\right)}{\left(\frac{m_{3}\gamma_{th}}{m_{s3}{\bar{\gamma }}_{3}b}\right)}^{m_{3}}\frac{1}{\Gamma \left(m_{2}\right)}{\left(\frac{m_{2}}{a{\bar{\gamma }}_{2}}\right)}^{m_{2}}\\ \;\;\;\;\;\;\;\;\;\cdot \int_{0}^{\infty }\gamma_{2}^{-m_{3}+m_{2}-1}G_{2,2}^{2,1}\left(\frac{m_{s3}{\bar{\gamma }}_{3}b\gamma_{2}}{m_{3}\gamma_{th}}|\begin{array}{c} 1,1+m_{3}\\ m_{3},m_{3}+m_{s3} \end{array}\right)G_{0,1}^{1,0}\left(\frac{m_{2}}{a{\bar{\gamma }}_{2}}\gamma_{2}|\begin{array}{c} -\\ 0 \end{array}\right)d\gamma_{2}. \end{array}$$

Further, with the help of ([46], (07.34.21.0011.01)), the previous integral is solved in the exact closed-form, and *I*_1_ is represented as the following form:

(A9) $$\begin{array}{cc} I_{1} & =\frac{1}{\Gamma \left(m_{2}\right)\Gamma \left(m_{3}+m_{s3}\right)B\left(m_{3},m_{s3}\right)}\\ & \cdot {\left(\frac{m_{2}m_{3}\gamma_{th}}{a{\bar{\gamma }}_{2}m_{s3}{\bar{\gamma }}_{3}b}\right)}^{m_{2}}G_{2,3}^{2,2}\left(\frac{m_{2}m_{3}\gamma_{th}}{a{\bar{\gamma }}_{2}m_{s3}{\bar{\gamma }}_{1}b}|\begin{array}{c} 1-m_{2},1-m_{2}-m_{s3}\\ 0,m_{3}-m_{2},-m_{2} \end{array}\right). \end{array}$$

Furthermore, by substituting Equations (A4) and (1) into Equation (A3), with appropriate indexed parameters, the expression of *I*_2_ is rewritten as

(A10) $$\begin{array}{c} I_{2}=\frac{1}{\Gamma \left(m_{2}\right)\Gamma \left(m_{3}+m_{s3}\right)B\left(m_{3},m_{s3}\right)}{\left(\frac{m_{3}\gamma_{th}}{m_{s3}{\bar{\gamma }}_{3}b}\right)}^{m_{3}}{\left(\frac{m_{2}}{a{\bar{\gamma }}_{2}}\right)}^{m_{2}}\\ \;\;\;\;\;\;\;\;\;\cdot \int_{0}^{\gamma_{th}}\gamma_{2}^{-m_{3}+m_{2}-1}G_{2,2}^{1,2}\left(\frac{m_{3}\gamma_{th}}{m_{s3}{\bar{\gamma }}_{3}b\gamma_{2}}|\begin{array}{c} 1-m_{3},1-m_{3}-m_{s3}\\ 0,-m_{3} \end{array}\right)e^{-\frac{m_{2}}{a{\bar{\gamma }}_{2}}\gamma_{2}}d\gamma_{2}. \end{array}$$

The integral in *I*_2_ cannot be obtained in the closed form, following the approach used for solving the integral in *I*_1_. Since the argument of the exponential function is a small value (i.e., a>>1), by expanding the exponential function into a series ([46], (01.03.06.0002.01)) and taking into account only the first term, without loss of generality, we obtain the following approximate form:

(A11) $$\begin{array}{c} I_{2}≅\frac{1}{\Gamma \left(m_{2}\right)\Gamma \left(m_{3}+m_{s3}\right)B\left(m_{3},m_{s3}\right)}{\left(\frac{m_{3}\gamma_{th}}{m_{s3}{\bar{\gamma }}_{3}b}\right)}^{m_{3}}{\left(\frac{m_{2}}{a{\bar{\gamma }}_{2}}\right)}^{m_{2}}\\ \;\;\;\;\;\;\cdot \int_{0}^{\gamma_{th}}\gamma_{2}^{-m_{3}+m_{2}-1}G_{2,2}^{1,2}\left(\frac{m_{3}\gamma_{th}}{m_{s3}{\bar{\gamma }}_{3}b\gamma_{2}}|\begin{array}{c} 1-m_{3},1-m_{3}-m_{s3}\\ 0,-m_{3} \end{array}\right)d\gamma_{2}. \end{array}$$

Now, the previous integral can be solved utilizing ([46], (07.34.21.0084.01)), and the integral *I*_2_ becomes

(A12) $$\begin{array}{c} I_{2}≅\frac{\gamma_{th}^{m_{2}-m_{3}}}{\Gamma \left(m_{2}\right)\Gamma \left(m_{3}+m_{s3}\right)B\left(m_{3},m_{s3}\right)}{\left(\frac{m_{3}\gamma_{th}}{m_{s3}{\bar{\gamma }}_{3}b}\right)}^{m_{3}}{\left(\frac{m_{2}}{a{\bar{\gamma }}_{2}}\right)}^{m_{2}}\\ \;\cdot G_{3,3}^{2,2}\left(\frac{m_{s3}{\bar{\gamma }}_{3}b}{m_{3}}|\begin{array}{c} \begin{array}{ccc} 1-m_{2}+m_{3}, & 1, & 1+m_{3} \end{array}\\ \begin{array}{ccc} m_{3}, & m_{2}+m_{s3}, & m_{3}-m_{2} \end{array} \end{array}\right). \end{array}$$

Finally, the probability $\mathrm{Pr}\left\{\gamma_{3}\leq \gamma_{th},\gamma_{2}>\gamma_{th}\right\}$ is obtained by substituting Equations (A9) and (A12) into Equation (A1) in the form of Equation (19).
